# Efficient target cleavage by Type V Cas12a effectors programmed with split CRISPR RNA

**DOI:** 10.1093/nar/gkab1227

**Published:** 2021-12-24

**Authors:** Regina Shebanova, Natalia Nikitchina, Nikita Shebanov, Vladimir Mekler, Konstantin Kuznedelov, Egor Ulashchik, Ruslan Vasilev, Olga Sharko, Vadim Shmanai, Ivan Tarassov, Konstantin Severinov, Nina Entelis, Ilya Mazunin

**Affiliations:** Center of Life Sciences, Skolkovo Institute of Science and Technology, Moscow 143026, Russia; Center of Life Sciences, Skolkovo Institute of Science and Technology, Moscow 143026, Russia; UMR7156 – Molecular Genetics, Genomics, Microbiology, University of Strasbourg and Centre National de la Recherche Scientifique (C.N.R.S.), Strasbourg 67000, France; Center of Life Sciences, Skolkovo Institute of Science and Technology, Moscow 143026, Russia; Waksman Institute of Microbiology, Rutgers The State University of New Jersey, Piscataway 08854, USA; Waksman Institute of Microbiology, Rutgers The State University of New Jersey, Piscataway 08854, USA; Laboratory of Bioconjugate Chemistry, Institute of Physical Organic Chemistry, National Academy of Science of Belarus, Minsk 220072, Belarus; Kurchatov Genomics Center, National Research Center “Kurchatov Institute”, Moscow 123098, Russia; Faculty of Biology, Lomonosov Moscow State University, Moscow 119991, Russia; Laboratory of Bioconjugate Chemistry, Institute of Physical Organic Chemistry, National Academy of Science of Belarus, Minsk 220072, Belarus; Laboratory of Bioconjugate Chemistry, Institute of Physical Organic Chemistry, National Academy of Science of Belarus, Minsk 220072, Belarus; UMR7156 – Molecular Genetics, Genomics, Microbiology, University of Strasbourg and Centre National de la Recherche Scientifique (C.N.R.S.), Strasbourg 67000, France; Center of Life Sciences, Skolkovo Institute of Science and Technology, Moscow 143026, Russia; Waksman Institute of Microbiology, Rutgers The State University of New Jersey, Piscataway 08854, USA; Center for Precision Genome Editing and Genetic Technologies for Biomedicine, Institute of Gene Biology, Russian Academy of Sciences, Moscow 119334, Russia; UMR7156 – Molecular Genetics, Genomics, Microbiology, University of Strasbourg and Centre National de la Recherche Scientifique (C.N.R.S.), Strasbourg 67000, France; Center of Life Sciences, Skolkovo Institute of Science and Technology, Moscow 143026, Russia

## Abstract

CRISPR RNAs (crRNAs) that direct target DNA cleavage by Type V Cas12a nucleases consist of constant repeat-derived 5′-scaffold moiety and variable 3′-spacer moieties. Here, we demonstrate that removal of most of the 20-nucleotide scaffold has only a slight effect on *in vitro* target DNA cleavage by a Cas12a ortholog from *Acidaminococcus* sp. (AsCas12a). In fact, residual cleavage was observed even in the presence of a 20-nucleotide crRNA spacer moiety only. crRNAs split into separate scaffold and spacer RNAs catalyzed highly specific and efficient cleavage of target DNA by AsCas12a *in vitro* and in lysates of human cells. In addition to dsDNA target cleavage, AsCas12a programmed with split crRNAs also catalyzed specific ssDNA target cleavage and non-specific ssDNA degradation (collateral activity). V-A effector nucleases from *Francisella novicida* (FnCas12a) and *Lachnospiraceae bacterium* (LbCas12a) were also functional with split crRNAs. Thus, the ability of V-A effectors to use split crRNAs appears to be a general property. Though higher concentrations of split crRNA components are needed to achieve efficient target cleavage, split crRNAs open new lines of inquiry into the mechanisms of target recognition and cleavage and may stimulate further development of single-tube multiplex and/or parallel diagnostic tests based on Cas12a nucleases.

## INTRODUCTION

**Figure 1.**
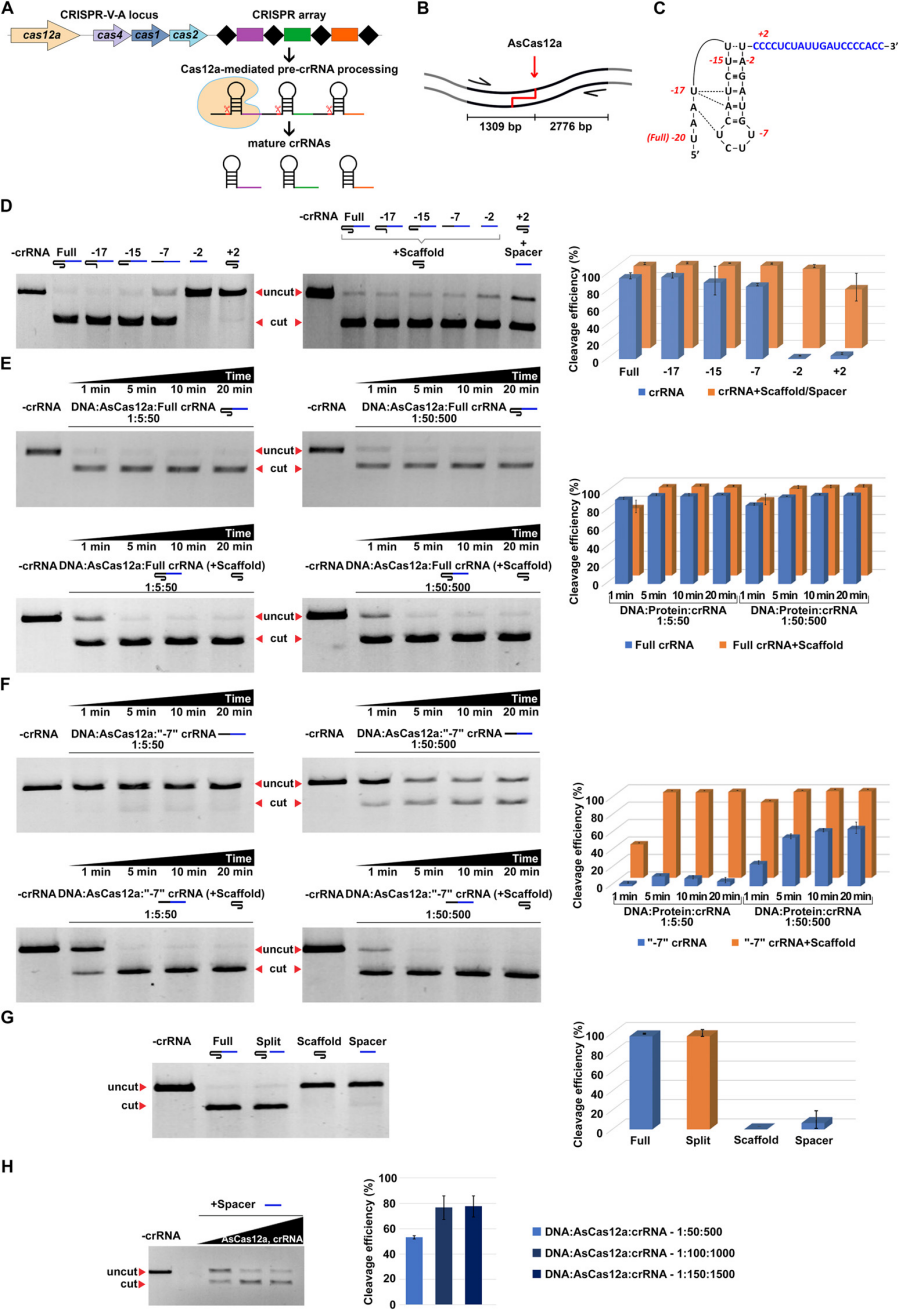
Addition of scaffold RNA stimulates target DNA cleavage by AsCas12a programmed with 5′ truncated crRNAs. (**A**) Schematic representation of Cas12a-mediated pre-crRNA processing. A subtype V-A CRISPR-Cas locus organization is shown (22). Genes coding for proteins responsible for interference (Cas12a) and adaptation (Cas4, Cas1, Cas2) are labeled and shown by colored arrows. The CRISPR array consists of spacers (colored rectangles) separated by repeats (black rhombi). Processing of the CRISPR array transcript (pre-crRNA) is carried out at the repeats by the Cas12a nuclease. (**B**) Schematics of the *in vitro* target DNA cleavage assay. A fragment of DNA containing the target site complementary to the crRNA spacer is amplified with PCR primers (indicated by arrows). Cleavage by AsCas12a programmed with crRNA recognizing the target is indicated by a red arrow and results in the appearance of cleavage products of indicated lengths. (**C**) The crRNA used for target recognition and cleavage. The scaffold moiety (black font) adopts a pseudoknot structure (12); the spacer sequence is highlighted in blue-coloured font. The numbers in red indicate the 5′-terminal nucleotides of truncated variants of crRNA tested. In the +2 variant, 18 nucleotides from the 3′-end of the spacer were eliminated. (**D**) Results of *in vitro* target cleavage assay with AsCas12a programmed with truncated crRNAs (indicated above the gel). The reactions contained 10 nM of the target, 500 nM of recombinant AsCas12a, and 5 μM of indicated crRNAs (a 1:50:500 ratio). Reactions were incubated 30 min at 37°C and products were resolved by agarose gel electrophoresis. Only the uncut target and one of the cleavage products (2776 bp) are shown. In the rightmost gel reactions were supplemented with 5 μM of scaffold or spacer RNA, as indicated. The results were quantified using ImageLab software. Mean cleavage efficiencies from four independent experiments and standard deviations are shown. (**E**,**F**) Kinetics and concentration dependence of target cleavage by AsCas12a programmed with full-sized (**E**) or –7 (**F**) crRNAs. Reactions proceeded for 1, 5, 10 and 20 minutes and were carried out at the indicated target:AsCas12a:crRNA ratios. Gels at the bottom of each panel show the results of cleavage assays conducted in the presence of equimolar amounts of scaffold crRNA moiety. Mean cleavage efficiencies and standard deviations from three independent experiments are shown on the right. (**G**) Target DNA cleavage by AsCas12a programmed with 3 μM full-sized, split, or separate scaffold and spacer crRNA moieties at a 1:30:300 target:AsCas12a:RNA ratio. ‘Split’ reactions contained both the scaffold and spacer RNAs. Mean cleavage efficiencies and standard deviations from three independent experiments are shown on the right. (**H**) Cleavage by AsCas12a programmed with increasing concentrations (5, 10 and 15 μM) of spacer RNA, corresponding to 1:50:500, 1:50:1000 and 1:50:1500 DNA:AsCas12a:crRNA ratios. Mean cleavage efficiencies and standard deviations calculated from three independent experiments are shown.

The CRISPR-Cas systems provide adaptive immunity to bacteria and archaea (1–3). They consist of CRISPR (clustered regularly interspaced short palindromic repeats) arrays and CRISPR-associated (Cas) proteins (Figure 1A). CRISPR arrays are composed of identical repeats separated by variable spacer sequences acquired from mobile genetic elements through the function of site-specific integrases composed of Cas1, Cas2, and, in some cases, Cas4 proteins. CRISPR arrays are transcribed into pre-CRISPR RNAs, which are processed at the repeat sequences generating short CRISPR RNAs (crRNAs). Individual crRNAs program Cas effector nucleases to recognize and cleave nucleic acids containing protospacers—sequences complementary to spacers. To prevent self immunity that could arise through recognition of CRISPR array spacers from which crRNAs are derived, many effectors require, in addition to crRNA spacer-target protospacer complementarity, the presence of a short Protospacer Adjacent Motif (PAM) that is specifically recognized by the effector protein. Since there is no PAM sequence in the repeat, targeting of the array is prevented.

At present, two classes, six types and numerous subtypes of CRISPR-Cas systems have been described and some have been harnessed for genomic editing (4–6). AsCas12a, a class 2 Type V-A effector nuclease from *Acidaminococcus* sp., has been used to edit genomes in a number of organisms, including human cells (7–10). The crRNAs used by the AsCas12a effector consist of two nearly equally sized moieties, a repeat-derived scaffold (also referred to as the 5′-handle), which is identical for different crRNAs, and a variable spacer or guide segment complementary to target DNA sequences (Figure 1A). Like other V-A effectors, AsCas12a alone is able to process pre-crRNA transcripts into mature crRNAs (11).

To date, structures of three type V-A Cas effector proteins, AsCas12a (12), LbCas12a from a *Lachnospiraceae bacterium* (13), and FnCas12a from *Francisella novicida* (14) charged with cognate crRNAs and bound to target DNA have been determined. These structures revealed that the crRNA scaffold adopts a pseudoknot structure, rather than a simple stem–loop predicted from its nucleotide sequence (12). The Cas12a proteins interact with this pseudoknot (13). Cas12a/crRNA complexes recognize T-rich PAMs located at the 3′ ends of their targets and induce a staggered double-stranded break within the region complementary to crRNA spacer (12,15). Unlike other effectors, the Cas12a enzymes can also catalyse specific cleavage of single-stranded targets complementary to crRNA spacer (a single cut is introduced in this case) and nonspecific (collateral) ssDNA degradation stimulated by the recognition of either single- or double-stranded targets (16,17).

Previous studies analyzed crRNA structure requirements for DNA cleavage by Cas12a nucleases (13,14,18). It was demonstrated that extending the length of crRNA at the 5′ (i.e. scaffold) end enhances mammalian gene editing efficiency by AsCas12a (19). This approach was used to extend AsCas12a crRNAs with switchable domains activated by trigger RNAs via strand displacement, which can be adapted for transcriptional control of gene expression in *Escherichia coli* (20). On the other hand, shortening of the scaffold and/or spacer crRNA moiety decreased the FnCas12a and AsCas12a cleavage activities (15,18). It was also shown that the FnCas12a nuclease activity depends on the secondary structure of crRNA scaffold and is sensitive to nucleotide changes in this region (15). In this work, we studied the effect of truncation and splitting of crRNAs on *in vitro* target DNA cleavage by Cas12a nucleases. We demonstrate that the cleavage activity persists after substantial reduction of the crRNA scaffold length and can even be detected upon complete removal of the scaffold, i.e. in the presence of a 20-nucleotide RNA matching the spacer moiety only. Further, we show that the residual AsCas12a nuclease activity charged with spacer RNA can be brought to levels typical for effectors charged with full-sized crRNA by the *in trans* addition of a 20-base scaffold RNA. This is observed both in a pure *in vitro* system and in human cell lysates. When programmed with cognate split crRNAs AsCas12a, and two other V-A effectors tested—FnCas12a, and LbCas12a—demonstrated specific dsDNA target cleavage, specific ssDNA target cleavage, and non-specific collateral degradation of ssDNA stimulated by target recognition. We conclude that the ability to use split crRNAs is a general property of V-A effectors. Though split crRNAs are functional at higher concentrations than full-sized crRNAs, we propose that the use of split crRNAs for specific recognition and cleavage of DNA templates opens ways for future mechanistic research and practical applications of Cas12a effectors that require multiplexing and/or simultaneous interrogation of multiple targets.

## MATERIALS AND METHODS

### Cas nucleases

AsCas12a and SpCas9 nucleases were expressed from plasmid vectors (Addgene, Plasmids #90095, #62374) and purified from *E. coli* expression strain BL21-DE3 as described (8) with modifications. Briefly, the lysate of cells was loaded onto a HiTrap Chelating HP column (GE Healthcare) equilibrated with a buffer containing 20 mM imidazole. The recombinant protein was eluted with a buffer containing 500 mM imidazole, mixed with TEV protease (for AsCas12a), and dialyzed at 4°C for 12 h. Then the proteins were concentrated using Amicon Ultra 10K filter (Millipore) and applied to HiLoad Superdex 200 16/60 column (GE Healthcare). Fractions containing either AsCas12a or SpCas9 were collected, concentrated and then snap-frozen in liquid nitrogen in small aliquots that were stored at –80°C until use. The protein concentration was measured using Qubit Protein Assay Kit (Thermo Fisher) according to the manufacturer′s instructions. The protein quality was tested by SDS-PAGE gel electrophoresis and visualized after Coomassie blue staining. LbCas12a and FnCas12a were purchased from OriGene Technologies and Biocat GmbH, respectively.

### *In vitro* DNA cleavage assay

All RNAs and DNA oligonucleotides used in this study are listed in Supplementary Table S1 provided by PRIMETECH ALC and IDT. The chemical synthesis of modified crRNA molecules is described in Supplementary Data.

*In vitro* cleavage assays were carried out in 30 μl in 1× NEB2.1 buffer (NEB). DNA substrates for *in vitro* cleavage represent fragments of DNA amplified by PCR in Q5^®^ High-Fidelity 2X Master Mix (M0492L, NEB). As a template, genomic DNA isolated from T-REx cell line (T-REx™-293 Cell Line, Invitrogen, kat. # R71007) by GeneJET Genomic DNA Purification Kit (K0722, Thermo Scientific™) was used. DNA concentration was measured by Qubit 3.0 fluorometer using Qubit DNA BR Assay Kit (Q32853, Thermo Scientific™). The standard reaction containing 10 nM of DNA template, 500 nM of the nuclease, and various amounts of crRNA. Reactions were incubated for 30 min at 37°C and stopped by the addition of 40 μg of Proteinase K (Promega) and additional incubation at 37°C for 15 min. Analysis of cleavage products was performed by electrophoresis in 1% agarose gel in 1× TBE buffer. EtBr staining gels were visualized by Gel Doc EZ Imager (Bio-Rad) and quantified with Image Lab™ Software 6.0.1 (Bio-Rad).

To assess specific and non-specific DNase activity of AsCas12a, LbCas12a and FnCas12a both ssDNA and dsDNA templates were used. Bacteriophage φX174 virion ssDNA and RF IdsDNA were purchased from NEB. DNA oligonucleotide duplexes were formed by mixing labelled strand and unlabeled complementary strand at a molar ratio of 1:1.5 in Nuclease-Free Duplex Buffer (IDT), heating for 2 min at 95°C and slow cooling to 20°C. The complexes were reconstituted in reaction buffer by mixing one of the Cas12a nucleases and corresponding full-sized crRNA or split crRNA at a 1:2 molar ratio followed by 20-min incubation at RT. Complexes were kept on ice before use. All cleavage reactions were carried out in 10 μL buffer containing 40 mM Tris–HCl, pH 7.5, 50 mM NaCl, 5 mM MgCl_2_, 0.5 mM TCEP, 0.1 mg/mL BSA, at 37°C for 30 min. Nonspecific digestion of 10 nM φX174 DNA was performed by 25 nM Cas12a–crRNA complex in the presence of 60 nM target ssDNA or dsDNA (activators). Reactions were quenched with Gel Loading Dye Purple (NEB) containing 0.4 mg/mL heparin and cleavage products were resolved by 1% agarose gel electrophoresis pre-stained with EtBr.

Fluorescein labelled ss/dsDNA substrates were used at 40 nM concentration to demonstrate specific cleavage activity of complexes at 150 nM concentration and nonspecific activity at 50 nM concentration. 60 nM ssDNA DNA was used as an activator. The reactions were terminated by the addition of formamide-containing loading buffer with 0.4 mg/ml heparin and heated in a boiling water bath for 1 min. Products were resolved by urea-denaturing 10% PAGE. Gels were imaged by Amersham Typhoon scanner.

### Fluorescence assay of beacon DNA binding

Fluorescence measurements were performed using a QuantaMaster QM4 spectrofluorometer (PTI) in binding buffer (20 mM Tris–HCl (pH 7.5), 50 mM NaCl, 0.1 mM DTT and 5 mM MgCl_2_) at 25ºC. Double-stranded beacon DNA was formed by mixing equimolar amounts of synthetic complementary strands (final concentrations were within low μM range) in a buffer containing 20 mM Tris, pH 7.5, 100 mM NaCl by heating for 2 min at 90ºC and slowly cooling the reactions down to 20ºC. The beacon DNA construct shown in Figure 3A was previously described in (21). The protospacer adjacent motif (PAM)-distal ends of the beacon target and non-target strands were labelled with fluorescein and Iowa BlackFQ, respectively. Final assay mixtures (800 μl) contained 40 nM of one of the Cas12a nucleases, 1 nM Cas12a beacon and 60 nM of corresponding full-sized crRNA or its fragments (scaffold and spacer). In control experiment, 100 nM spacer was added in the absence of AsCas12a. Time-dependent fluorescence changes were monitored after the addition of a negligible volume of Cas12a beacon to a cuvette followed by manual mixing; the mixing dead-time was 15 s.

### DNA cleavage assay in hAsCas12a expressing cell lysate

The hAsCas12a gene was introduced into human HEK 293 T-REx cell line nuclear genome using the Flp-In™ System (Flp-In™ T-REx™-293 Cell Line, Invitrogen) allowing controlled expression by the tet-on system. Successful integration was monitored by antibiotic selection with hygromycin B (100 μg/mL, Invivogen) and blasticidin (5 μg/ml, Invivogen). Expression of AsCas12a nuclease in the stable HEK 293 T-REx cell line was induced by 100 ng/ml tetracycline for 24 h and assayed by Western blotting using FLAG-tag specific antibodies (Sigma-Aldrich, F1804).

Cells with inducible expression of the AsCas12a nuclease were cultured in 6-well plate at 37°C, 5% CO_2_ in standard essential modified Eagle′s medium (EMEM) containing 4.5 g/l d-glucose, and 5 mg/ml uridine supplemented with 10% fetal bovine serum. The expression of the nuclease was activated at 60–80% confluency by the addition of tetracycline to a final concentration 100 ng/ml. Twenty four hours post-induction, cells were washed with 1× PBS, resuspended in 500 μl lysis buffer (20 mM HEPES–KOH pH 7.5, 100 mM KCl, 5 mM MgCl_2_, 1 mM DTT, 5% glycerol, 0.1% Triton X-100 and Protease Inhibitor cocktail) and rotated for 10 min at 4°C. To remove cell debris, lysates were centrifuged for 10 min at 10 000g, 4°C and the supernatant was snap-frozen in small aliquots and stored at –80°C until use.

The cleavage reactions were carried out in the cleavage buffer (20 mM HEPES–KOH pH 7.5, 100 mM KCl, 5 mM MgCl_2_, 1 mM DTT, 5% glycerol) containing 4 nM of PCR-obtained DNA template (oligonucleotide primers are listed in Supplementary Table S1) and various amounts of crRNA 30 min at 37°C and stopped by the addition of 40 μg of Proteinase K (Promega). After 15-min incubation at 37°C products were purified with NucleoSpin PCR Clean-up kit (Macherey-Nagel). Analysis of cleavage products was performed by electrophoresis in 1% agarose gel in 1× TAE buffer. EtBr-stained gels were visualized by Hero Lab Transilluminator UVT-28M (Herolab GmbH) and quantified with Image Lab™ Software 6.0.1 (Bio-Rad).

### T7E1 assays

HEK 293 T-REx hAsCas12a cells were cultured in 100 mm dishes and transfected with 4 μg of full-sized crRNA or 2 μg of each moiety of split RNA (scaffold and spacer) targeting *DNMT1* gene (RNAs sequences can be found in Supplementary Table S1). Expression of AsCas12a nuclease was induced by 100 ng/mL tetracycline. Cells were collected 24 h after transfection, and genomic DNA was isolated using GeneJET Genomic DNA Purification Kit (K0722, Thermo Scientific™) according to the manufacture′s protocol. Genomic fragments were amplified with Phusion High Fidelity polymerase (New England Biolabs) (oligonucleotide primers are listed in Supplementary Table S1). PCR reactions were cleaned up with Qiagen Gel purification Kit (Qiagen). Hybridization of PCR products was carried out in 19 μl reaction mix containing 1000 ng DNA, 2 μl Nebuffer 2 (NEB) and 1 μl of T7 Endonuclease (NEB) with following conditions: denaturation at 95°C for 5 min, annealing at 95–85°C –2°C/s, at 85–25°C –0.1°C/s. Reactions were incubated for 1 h at 37°C. Products were seprated on a 2% agarose gel and visualised with Ethidium Bromide.

## RESULTS

### 5′ truncated and split crRNAs, as well as spacer-only RNAs support specific target cleavage by AsCas12a *in vitro*

Recombinant AsCas12a was tested with a set of chemically synthesized crRNAs truncated from the 5′-end, i.e., harbouring a scaffold moiety of different lengths and a constant 20-nt spacer moiety (versions –17, –15, –7 and –2, Figure 1С) in an *in vitro* DNA cleavage assay. As a cleavage substrate, a PCR-amplified fragment containing an asymmetrically located target sequence complementary to crRNA spacer with upstream consensus PAM was used (Figure 1B). The truncated crRNA versions used here match those studied by Zetsche *et al.* (15). Full-sized crRNA with a 20-nt scaffold was used as a positive control. An RNA that consisted of a full-sized scaffold extended by just two extra nucleotides of the spacer (version +2) was also tested. The reaction products were separated by gel electrophoresis, visualized by ethidium bromide staining and the extent of DNA substrate cleavage was quantified as described in the Materials and Methods. As can be seen from the leftmost gel in Figure 1D, the AsCas12a nuclease was surprisingly tolerant to 5′-truncations of the scaffold. At the conditions of the experiment, more than 80% target cleavage was observed in reactions containing full-sized, –17 and –15 crRNAs, and >60% of the DNA substrate was cleaved when the crRNA scaffold moiety was reduced to just seven nucleotides (version –7). Effectors charged with –2 and +2 crRNAs were inactive.

We were interested to determine whether the lack of activity of the –2 crRNA version could be complemented by the addition of scaffold RNA. Effectors charged with other crRNA versions were also tested in the presence of the scaffold. As can be seen from the rightmost gel in Figure 1D, the *in trans* addition of scaffold RNA stimulated target cleavage in the presence of –7 and –2 crRNA versions, which reached almost the same level as that seen with full-sized crRNA. Reciprocally, the addition of the 20-nt RNA spacer stimulated cleavage by the inactive effector charged with the +2 version (essentially, a scaffold RNA), which reached 80%.

We next investigated the kinetics and concentration dependence of target DNA cleavage by AsCas12a charged by either full-sized or truncated (version –7) crRNAs. As can be seen from Figure 1E, upper panels, in reactions containing full-sized crRNA, target DNA was cleaved ∼90% just after 1-min incubation at either 1:5:50 or 1:50:500 target DNA:AsCas12a:crRNA molar ratios and the addition of equimolar amounts of scaffold RNA did not affect target DNA cleavage in the presence of full-sized crRNA (Figure 1E, lower panels). In reactions containing the –7 crRNA in a 1:5:50 target DNA:AsCas12a:crRNA ratio target cleavage was undetectable. At a higher 1:50:500 ratio, partial target cleavage was observed (from 25 to 60% over the course of the reaction). Both the velocity and the extent of target cleavage were dramatically increased when reactions containing the –7 crRNA were supplemented with equimolar amounts of scaffold added *in trans*: the cleavage reaction was 40% complete after 1-minute incubation at low 1:5:50 target DNA:AsCas12a:crRNA ratio (98% after 5-min incubation) and 87% complete after 1-min incubation at the high 1:50:500 ratio (Figure 1F).

The results obtained with the +2 crRNA version supplemented with spacer RNA suggested that AsCas12a is able to locate and cleave its targets when supplemented with split crRNA containing separate scaffold and spacer moieties. Indeed, as can be seen from Figure 1G, the simultaneous addition of two crRNA moieties (a condition further referred to as ‘split crRNA’) induced specific cleavage of the target, which was as efficient as that induced by full-sized crRNA (∼95%) at the conditions of the experiment. Neither the spacer nor the scaffold RNAs taken separately caused substrate cleavage at these conditions. Efficient cleavage of cognate targets was also observed when split crRNAs with unrelated spacer sequences were tested (Supplementary Materials, Figure S1). Thus, split crRNA activity is not dependent on the sequence of the crRNA spacer moiety.

Because split crRNA/AsCas12a can cleave the DNA template quite efficiently, we wondered whether the spacer RNA moiety alone could also induce cleavage. We surmised that though no cleavage was obtained in the presence of spacer RNA in the experiment shown in Figure 1G, increasing the concentration of spacer RNA may allow the binding to the effector and target cleavage. An alternative scenario would be that the binding of the scaffold RNA is required for productive interaction with the spacer RNA by, for example, inducing a conformational change in the effector, and thus no target cleavage in the presence of spacer RNA shall be observed even at high concentrations. As can be seen from Figure 1H, increasing the concentration of spacer RNA from 5 to 15 μM led to dose-dependent DNA substrate cleavage by AsCas12a. These data demonstrate that AsCas12a charged with a 20-nt spacer RNA is capable of sequence-specific PAM-dependent cleavage of DNA substrates. This is possible only at high concentrations of RNA and a protein:RNA ratio of at least 1:20.

### Functional biochemical and biophysical analyses of split crRNA and spacer RNA interaction with the AsCas12a effector

**Figure 2.**
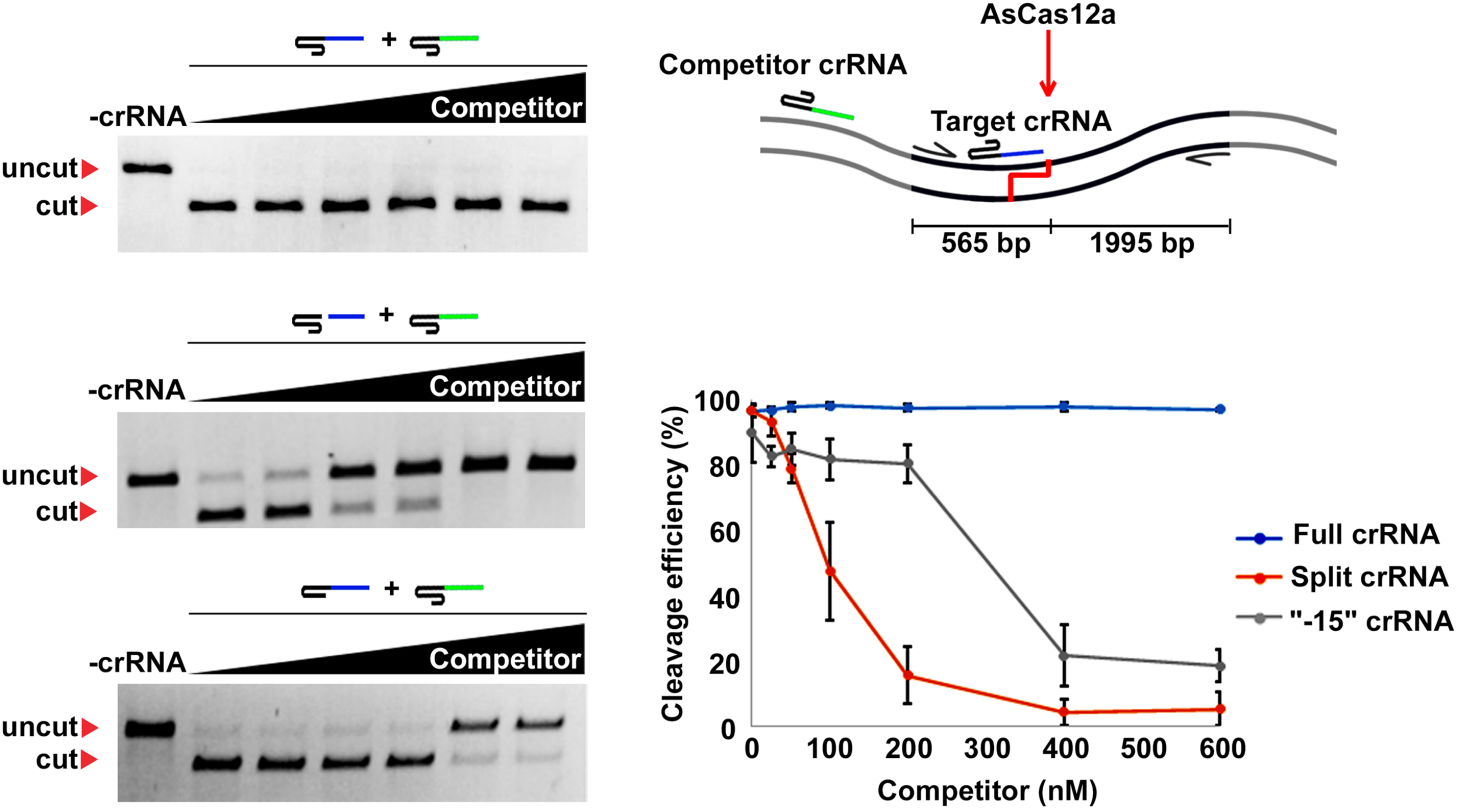
Effect of split crRNA on AsCas12a cleavage activity in presence of competitor crRNA. At top right, schematics of the target cleavage assay in the presence of competitor crRNA is shown. The assay is conducted as in Figure 1B but in the presence of a competitor crRNA that recognizes a target located outside of the amplified cleavage substrate. The spacer moieties of target and competitor crRNAs are shown in blue and green, correspondingly. Below, the results of cleavage assays with AsCas12a programmed with 500 nM of full-sized, split, or -15 crRNAs (a 1:5:50 target:AsCas12a:crRNA ratio) in the presence of increasing concentrations (0, 25, 50, 100, 200, 400, 600 nM) of competitor crRNA are presented; mean cleavage efficiencies and standard deviations calculated from three independent experiments are shown on the right.

To compare the binding of full-sized and split crRNA to AsCas12a, we performed *in vitro* DNA cleavage assay in the presence of increasing amounts of another crRNA (hereafter referred to as ‘crRNA competitor’) that targeted a sequence that was absent from the DNA cleavage substrate (Figure 2). The competitor was added to 30 nM AsCas12a together with full-sized, truncated, or split crRNA, followed by the addition of the target. As can be seen from Figure 2, the addition of up to 600 nM competitor had no effect on the cleavage of 10 nM target DNA by the effector charged with full-sized cognate crRNA present at 500 nM concentration. In contrast, cleavage by the effector charged with the truncated -15 crRNA was inhibited 50% in the presence of ∼300 nM competitor RNA, suggesting ∼2-fold reduction of affinity for AsCas12a compared to full-sized crRNA. Target cleavage by the effector charged with split crRNA was inhibited 50% in the presence of 100 nM competitor, indicating that split RNA affinity for the effector is reduced ∼5-fold compared to full-sized crRNA.

**Figure 3.**
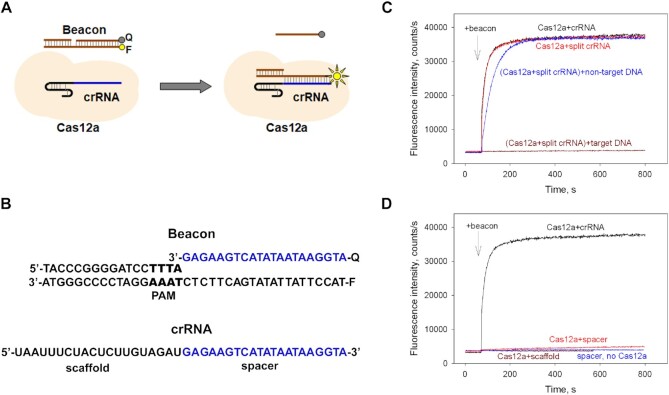
Analysis of the DNA target binding by the Cas12a loaded with split crRNA by the Cas beacon assay. (**A**) Principle of the Cas12a beacon assay. The circles labeled F and Q indicate the fluorophore and quencher. (**B**) Structures of the Cas12a beacon and of the crRNA used to program AsCas12a. The crRNA spacer and corresponding sequences of the beacon are highlighted in blue font, the PAM sequence is highlighted in bold font. (**C**) Time dependence of the increase in fluorescence upon the addition of 1 nM beacon to 40 nM AsCas12a loaded with 60 nM full-sized crRNA (black curve) or split crRNA (red curve) and effects of target (in brown) and non-target (in blue) DNA competitors on beacon binding to split crRNA-AsCas12a. (**D**) Time dependence of the increase in fluorescence upon the addition of 1 nM beacon to 40 nM AsCas12a loaded with 60 nM full-sized crRNA (in black), scaffold (in brown) or spacer (in red) crRNA fragments and to a control sample containing 100 nM spacer crRNA in the absence of AsCas12a (in blue).

To obtain further information on the split crRNA–AsCas12a complexes and their interactions with target DNA, a fluorescence Cas beacon method was implemented, following a strategy that was earlier used to monitor the interactions of various effector complexes with crRNAs and target DNAs (21,23,24). A schematic representation of the Cas12a beacon assay is shown in Figure 3A. The beacon is a fluorescently labeled derivative of target DNA. It is prepared by annealing three oligonucleotides to generate a 38 bp fragment of DNA comprising a 13 bp DNA upstream of PAM, a consensus AsCas12a PAM TTTA, and a 21-bp downstream segment recognized by the crRNA spacer. The PAM-distal ends of the beacon target and non-target strands are labeled with a fluorophore and a quencher, respectively. The baseline fluorescence intensity of a beacon is low due to quenching of the fluorescence label by a nearby quencher via the FRET mechanism. Binding of Cas12a–crRNA complex to the beacon should separate DNA strands bearing the fluorophore and the quencher, leading to a readily detectable increase in fluorescence intensity (Figure 3A). Indeed, upon the addition of the beacon shown in Figure 3B to AsCas12a pre-incubated with full-sized crRNA, the fluorescence intensity rapidly increased, reaching peak intensity in about a minute (Figure 3C). The kinetics of beacon binding to AsCas12a preincubated with split crRNA was identical to that observed with full-sized crRNA (Figure 3C), implying that the AsCas12a complexes with full-sized and split crRNAs have comparable DNA binding activities.

We also performed competition assays in which beacon binding to split crRNA–AsCas12a was monitored in the presence of either target DNA or non-target DNA that bore no complementarity with the crRNA sequence. Pre-incubation of split crRNA–Cas12a complex with target DNA for 15 min led to severe inhibition of beacon binding, while non-target DNA exerted only a modest effect (Figure 3C). This result indicated that split crRNA–AsCas12a formed a stable and specific complex with target DNA, thus corroborating data obtained in the DNA cleavage assay (Figure 1G). The fluorescence signal increased very slowly upon the beacon addition to a sample containing 40 nM AsCas12a and 60 nM spacer RNA (Figure 3D). The rate of signal increase in this experiment was about 500-fold lower than that observed when AsCas12a was programmed with full-sized or split crRNAs. This observation suggests that spacer RNA alone endows AsCas12a with a low affinity for target DNA. This fully corresponds with our finding that a spacer moiety is able to induce cleavage of the DNA substrate only at high concentrations (Figure 1H) that can not be reached in the beacon assay. No increase in beacon fluorescence intensity was observed in the presence of AsCas12a preincubated with the scaffold RNA or in the presence of spacer RNA without AsCas12a (Figure 3D). The latter control experiment shows that spacer RNA alone cannot anneal to the beacon and induce an increase in fluorescence under the conditions used.

**Figure 4.**
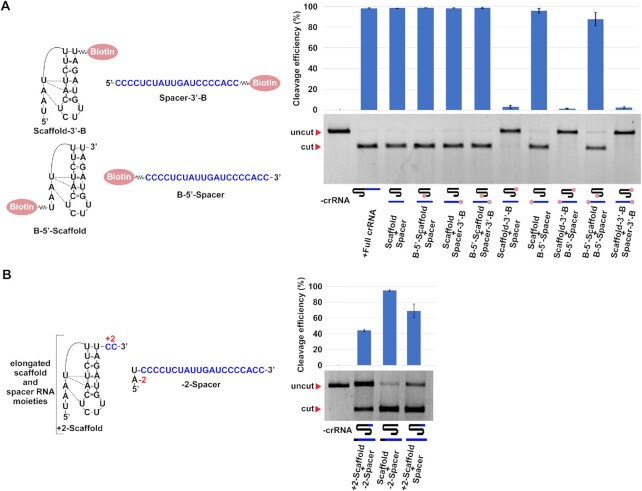
Effect of terminal modifications of spacer and scaffold RNAs on the activity of AsCs12a. (**A**) Scaffold and spacer crRNA moieties bearing either 5′- or 3′-terminal biotin modifications (shown as red ovals) were analyzed in the *in vitro* cleavage assay in all possible combinations. Mean cleavage efficiencies and standard deviations calculated from three independent experiments are shown. (**B**) As in (A), but using scaffold and spacer versions elongated by two non-complementary nucleotides (‘+2-Scaffold’ and ‘–2-Spacer’, correspondingly).

Data presented in Figure 1D shows that AsCas12a charged with spacer RNA and the +2 scaffold extended by two first nucleotides of the spacer moiety can recognize and cleave DNA targets. The result implies that split crRNA can accommodate extra nucleotides at the split site without compromising its function. To further investigate the importance of 5′- and 3′-extremities of the scaffold and spacer RNA moieties for split crRNA function we synthetized split versions bearing 5′- or 3′-terminal biotin modifications on the spacer and scaffold RNAs and tested them in the *in vitro* cleavage assay. Among all possible combinations tested, only the 3′-end biotin modification of the scaffold strongly decreased the DNA substrate cleavage efficiency (Figure 4A). This effect is surprising, given that the elongation of the scaffold 3′-end by two nucleotides had no strong effect (Figs. 1D and 4B), indicating that a non-nucleotide compound can disturb the scaffold-effector interaction. Biotin modification at the 5′-end of the spacer RNA did not influence the DNA substrate cleavage; however, a 2-nt extension of the spacer towards the junction site caused an additional decrease of cleavage in combination with the +2 version of the scaffold (Figure 4B).

**Figure 5.**
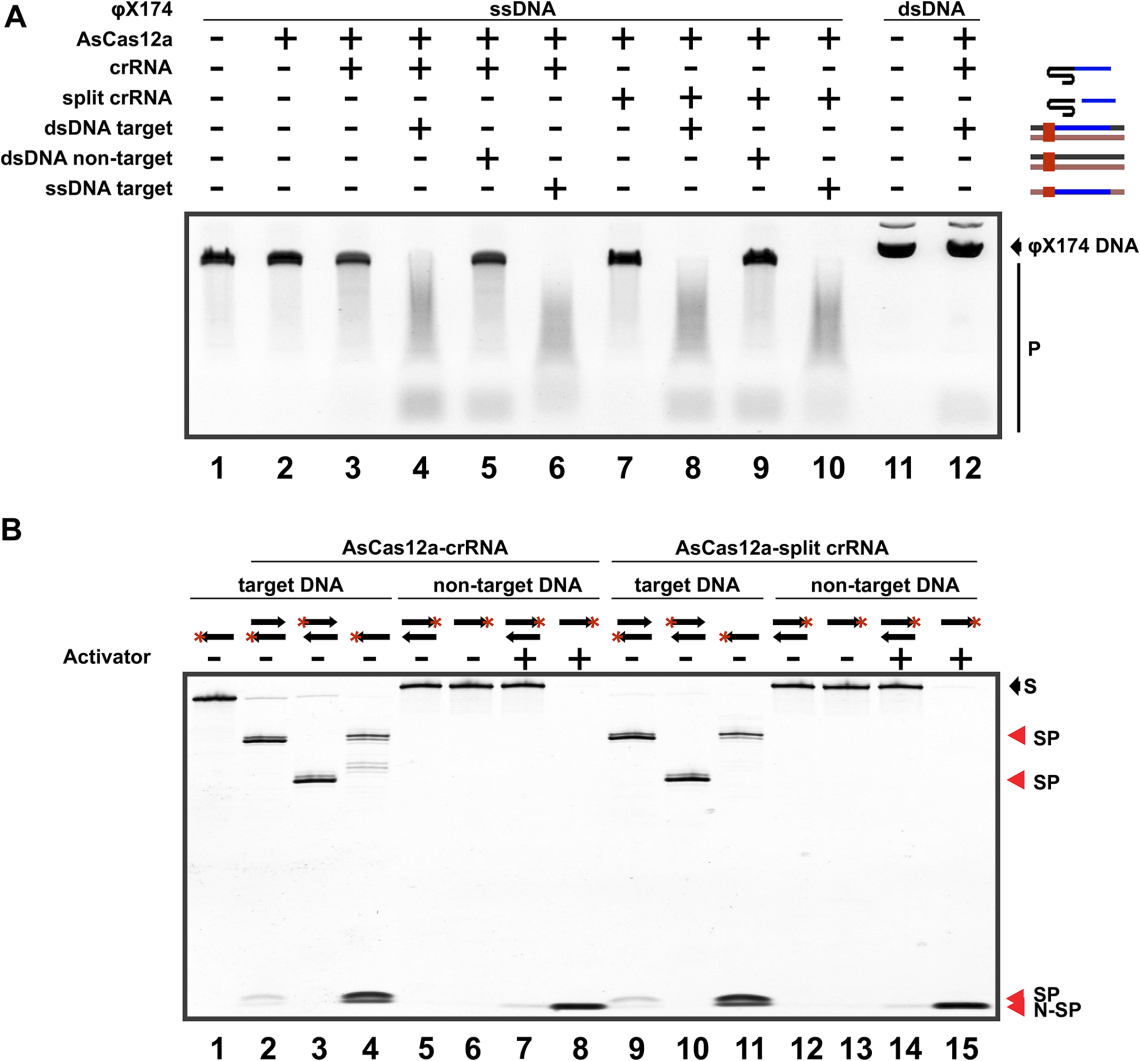
All known AsCas12a DNA cleavage activities are supported by split crRNA. (**A**) Non-specific ssDNA cleavage activity of AsCas12a against the φX174 ssDNA bearing no sequences matching crRNA was tested in the presence of full-sized and split crRNAs. Reactions were conducted in the presence of double-stranded DNA cleavage substrate matching the crRNA spacer, a single-stranded cleavage substrate with a sequence complementary to crRNA spacer and a functional PAM, or in the presence of double-stranded DNA bearing no sequences matching crRNA. Reaction products were resolved by agarose gel electrophoresis and revealed by ethidium bromide staining. Intact φX174 DNA and products of collateral cleavage activity are indicated. (**B**) Fluorescein-labeled (red asterisk) target and non-target DNA substrates, in the single-stranded or double-stranded form (shown schematically at the top of the gel and listed in Supplementary Table S1) were combined with AsCas12a programmed with the full-sized or split crRNA. 'Activator' is a non-labeled single-stranded oligonucleotide containing complementary to crRNA spacer and bearing a functional PAM. Reaction products were resolved by denaturing 10% PAGE. Substrate (‘S’) and specific (‘SP’) and non-specific (‘N-SP’) cleavage products are indicated on the right.

Cas12a enzymes catalyze not only specific dsDNA target cleavage but also specific cleavage of ssDNA targets and nonspecific (collateral) degradation of single-stranded DNA (ssDNA) induced by target cleavage (16,17). To check whether collateral cleavage activity of AsCas12a can be promoted by split crRNA, we pre-assembled AsCas12a with split crRNA and incubated with ssDNA or dsDNA targets in the presence of φX174 ssDNA that had no sequences matching crRNA. As can be seen from Figure 5A, cleavage of φX174 ssDNA was induced by recognition of either double-stranded and single-stranded targets by AsCas12a charged with control full-sized crRNA (lanes 4 and 6, correspondingly) or with split crRNA (lanes 8 and 10). No φX174 cleavage was observed in reactions without target DNA (lanes 3 and 7), or in the presence of non-target DNA (lanes 5 and 9).

To check for specific ssDNA cleavage activity of AsCas12a charged with split crRNA, 3′ terminally fluorescein-labeled target DNA substrates (see Supplementary Table S1) were used (17). Corresponding dsDNA substrates were used as controls. As can be seen from Figure 5B, AsCas12a charged with either full-sized or split crRNA cleaved double-stranded and single-stranded targets at the same positions (compare lanes 2 and 4, and lanes 9 and 11, respectively). Besides bands resulting from site-specific ssDNA cleavage, fast-mobility degradation products resulting from non-specific ssDNA cleavage activity described before (17) were seen both with full-sized and split crRNA charged effectors. We also showed that upon activation by a non-labeled target-strand substrate (activator), AsCas12a charged with either full-sized or split crRNA completely degraded labeled ss DNA (lanes 8 and 15), while leaving dsDNA intact (lanes 7 and 14), as was first reported in (16).

### Split crRNA is active in lysates of human cells expressing AsCas12a

**Figure 6.**
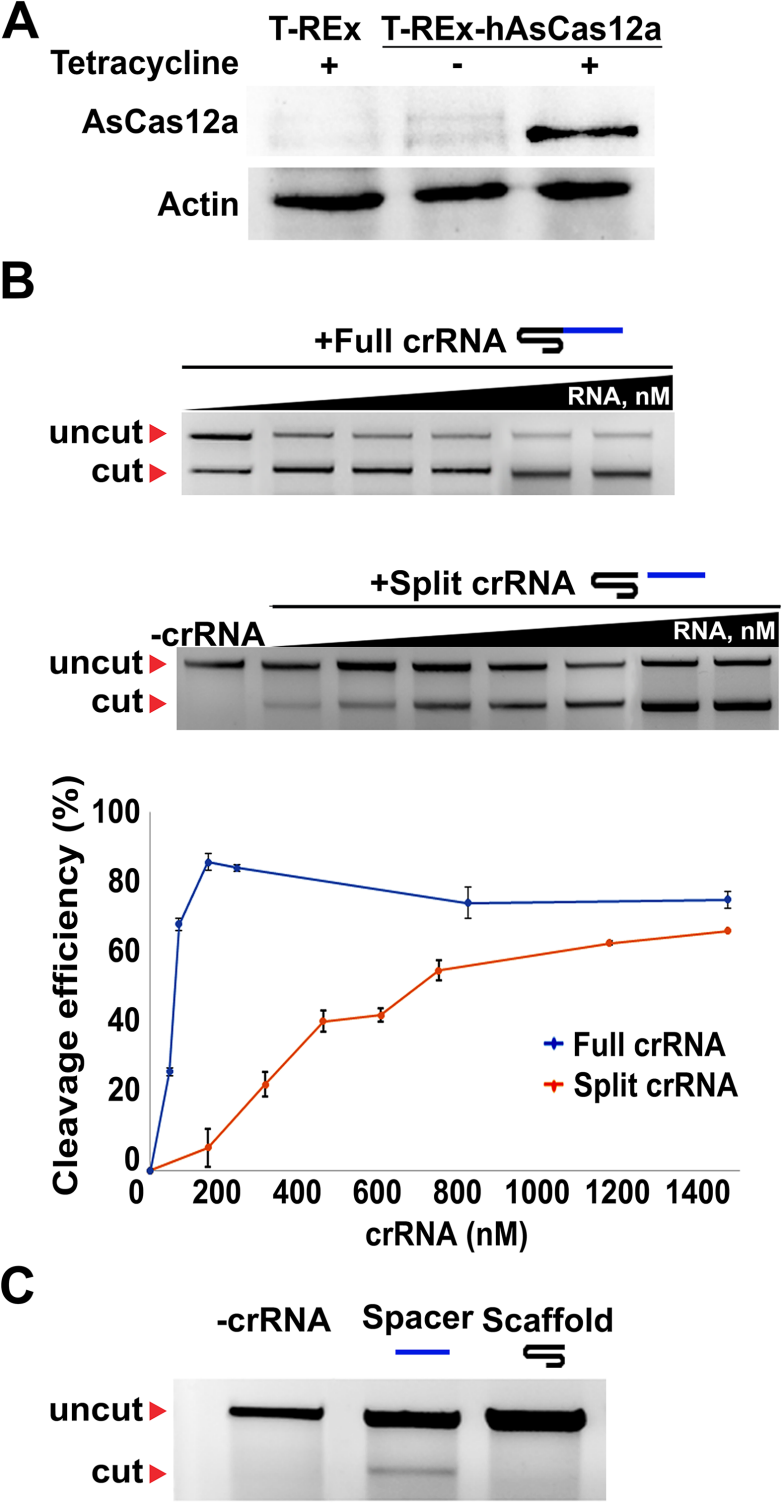
Split crRNA is active in lysates of human cells expressing AsCas12a. (**A**) Western blot analysis of hAsCas12a production in the stable HEK 293 T-REx-hAsCas12a cell line. FLAG-tag specific antibodies were used for hAsCas12a protein detection; actin specific antibodies were used for loading control. hAsCas12a gene transcription was induced by 100 ng/mL tetracycline. (**B**) Cleavage of the same DNA target is in Figure 1B was conducted in whole-cell lysates of induced cells in the presence of increasing concentrations of the full-sized or split crRNAs. Mean cleavage efficiencies and standard deviations calculated from three independent experiments are shown. (**C**) DNA template *in lysate* cleavage by spacer moiety of crRNA. DNA cleavage reaction in the presence of 27 μM spacer of scaffold crRNAs, incubated for 120 min at 37°C.

We next tested whether split crRNA can promote specific target cleavage in eukaryotic cell lysates. For this, a human cell line expressing hAsCas12a in a Tet-inducible manner was constructed (Figure 6А). Cleavage of target DNA was assayed in the lysate of induced cells supplemented with increasing concentrations of full-sized, split, or spacer crRNAs. The results revealed that cleavage activities of the hAsCas12a programmed with full-sized and split crRNAs are comparable at crRNA concentrations >800 nM but at lower concentrations the AsCas12a nuclease programmed by full-sized crRNA outperformed the one charged with the split version (Figure 6B). This corroborates data obtained with competitor crRNA (Figure 2) indicating lower affinity of split crRNA for AsCas12a. Nevertheless, the results demonstrate that split crRNA can induce specific cleavage of target DNA by AsCas12a nuclease expressed in human cells in the presence of all of the components of the cell lysate, most notably a large excess of cellular RNA. Target cleavage was only observed when >25 μM of spacer RNA was added to the lysate (Figure 6C), a concentration that is much higher than that needed for target cleavage in the pure system (Figure 1H). The result indicates that spacer RNA is poorly competing with cellular RNA present in the lysate. Scaffold RNA alone did not induce any DNA cleavage even at high concentrations, as expected.

We attempted to show specific cleavage induced by split RNA in human cells expressing hAsCas12a. However, at the conditions used, we did not detect target cleavage in cells transfected with split crRNA though ∼13% target cleavage was detected in cells transfected with corresponding full-sized crRNA (Supplementary Figure S2).

### Cas12a nucleases other than AsCas12a can use split crRNA

**Figure 7.**
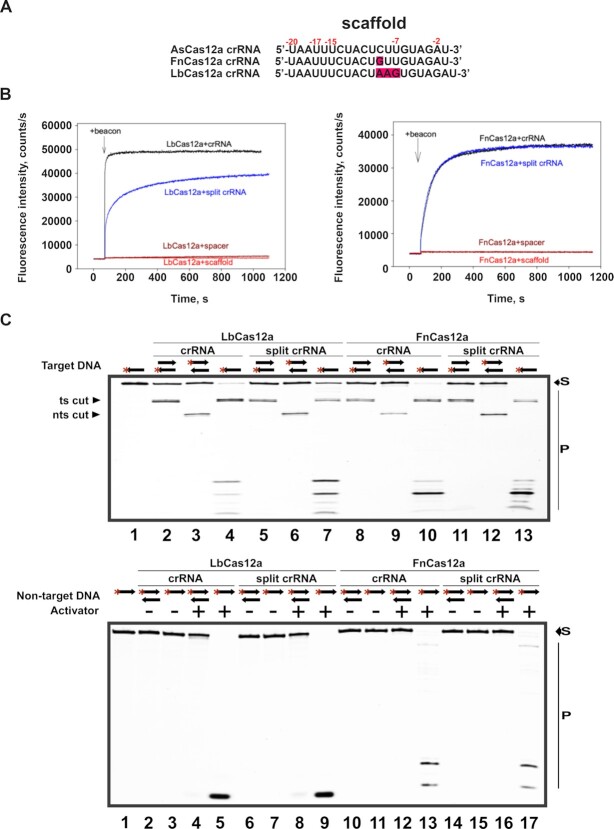
FnCas12a and LbCas12a effector nucleases programmed with cognate split crRNAs recognize and cleave their targets. (**A**) Sequences of the crRNA scaffold moiety for three V-A effector nucleases, As/Lb/FnCas12a (26). The nucleotide differences are highlighted in crimson. (**B**) Time dependence of beacon fluorescence increase upon the addition of the beacon to FnCas12a and LbCas12a loaded with full-sized crRNA (in black), split crRNA (in blue), scaffold (in red) or spacer (in brown) RNAs. The structures of the beacons and crRNAs are shown in Supplementary Table S1. (**C**) To demonstrate specific cleavage activity, fluorescein-labeled (red asterisk) target ssDNA (one arrow pictogram) and target dsDNA (two arrows pictogram) were digested by LbCas12a or FnCas12a in complex with full-sized (crRNA) or split crRNA. Activator-induced non-specific ssDNA cleavage activity was demonstrated using fluorescein-labeled non-specific ssDNA/dsDNA in the presence or absence of ssDNA activator. ‘Ts cut’ - target strand cut, and ‘nts cut’ - non-target strand cut, products of site-specific target DNA cleavage. Substrate (‘S’) and cleavage products (‘P’) were resolved by denaturing 10% PAGE.

We next determined whether Cas12a enzymes from *Lachnospiraceae bacterium* (LbCas12a) and *Francisella novicida* (FnCas12a) can be programmed for target recognition and cleavage with cognate split crRNAs. The DNA binding properties of FnCas12a and LbCas12a complexed with intact and split crRNAs were followed by measuring the kinetics of beacon binding to FnCas12a and LbCas12a preincubated with corresponding crRNAs (Figure 7A, B). We found that kinetics of beacon binding to FnCas12a preincubated with split crRNA was almost identical to that obtained with full-sized crRNA (Figure 7B), similarly to what was observed with AsCas12a (Figure 3C). In contrast, LbCas12a preincubated with split crRNA bound beacon significantly slower than LbCas12a preincubated with intact crRNA (Figure 7B). These data imply that the LbCas12a complex with split crRNA binds its targets less efficiently than the complex with full-sized crRNA. No change in beacon fluorescence was observed in experiments with FnCas12a and LbCas12a preincubated with the spacer or scaffold RNAs alone (Figure 7B), suggesting that spacer moieties interact with the effectors weakly or, alternatively, effectors complexes with spacer RNAs have low affinity for the targets.

The ability of split crRNAs to support target-activated nonspecific cleavage of ssDNA by LbCas12a and FnCas12a was also tested (Figure 7C and Supplementary Table S1). LbCas12a and FnCas12a in complex with split crRNA introduced site-specific cuts in dsDNA and ssDNA targets (Figure 7C). Both enzymes charged with split crRNAs were capable of target-activated degradation of non-specific ssDNA (Figure 7C) but not of dsDNA. We conclude that at least three distinct members of type V Cas12a nucleases, when programmed with split crRNA, can efficiently bind and cleave their targets and catalyze target-induced collateral degradation of ssDNA.

To determine whether the ability to use split RNAs might be a common property of Class 2 effectors, we performed *in vitro* target DNA cleavage assay using the most actively studied and widely used type II-А SpCas9 nuclease (1). The effector was charged with a single-guide RNA or its split version consisting of a 20-nt long spacer RNA complementary to the target and a longer RNA corresponding to the rest of sgRNA (referred to as ‘scaffold’ in Supplementary Figure S3). Only a very low level of DNA cleavage by SpCas9 was detected even at the highest concentrations of split RNA components (Supplementary Figure S3). It thus appears that, unlike Cas12a enzymes, SpCas9 and, by extension, other Cas9 nucleases, are unable to efficiently use split crRNA for target recognition and cleavage.

## DISCUSSION

The principal finding of this paper is the demonstration that different Type V Cas12a effectors can recognize and cleave their targets and perform target-activated collateral damage degradation of single-stranded DNA when charged with two short RNAs—one consisting of repeat-derived scaffold moiety of full-sized crRNA and another consisting of a spacer recognizing the target through complementary interactions. Crystal structures of AsCas12a/crRNA complexes revealed that the crRNA scaffold adopts a pseudoknot structure and is tightly bound in a groove between two AsCas12a monomers in a sequence-specific manner (12). The AsCas12a crRNA scaffold nucleotides U-10 and A-18 form a reverse Hoogsteen A:U base pair and participate in pseudoknot formation, which is additionally stabilized by non-canonical hydrogen bonding between U-10 and A-19 and between A-12, U-13 and U-17 (Figure 1C). The scaffold pseudoknot structure is ordered in both binary (Cas12a/crRNA) and ternary (Cas12a/crRNA/dsDNA) complexes; in contrast, the entire spacer moiety is disordered in the binary complex (13). It has been suggested that the initial binding of Cas12a to a PAM sequence in template DNA can induce conformational changes resulting in pre-ordering of the seed sequence comprising the first 8 scaffold-proximal nucleotides of the spacer (14). In the ternary complex, the heteroduplex between the target DNA strand and the spacer moiety of crRNA is accommodated in a positively charged central channel between the two lobes of the protein in a sequence-independent manner (9,14).

Previous studies analyzed the crRNA structure requirements for DNA cleavage by LbCas12a and FnCas12a nucleases (13,14). In the case of FnCas12a, deletion of 5′-terminal scaffold nucleotides A-19, A-18 and U-17 had little effect on DNA template cleavage efficiency (15). When a 5′-terminal deletion also removed U-16, the DNA cleavage was seriously affected, and further shortening of the scaffold completely abolished cleavage. Since A-19, A-18 and U-17 participate in the pseudoknot formation, deletion of these bases should turn the pseudoknot structure into a hairpin. Since in the hairpin U-16 forms a non-canonical pair with U-1, it appears that the hairpin structure rather than the pseudoknot is required for efficient target recognition and cleavage. Indeed, mutations that disrupt the helical part of the hairpin abolish target cleavage by FnCas12a. Our analysis shows that high concentrations of truncated –7 version of AsCas12a crRNA that is unable to form a hairpin are required for DNA cleavage. This is in agreement with previously published data on FnCas12a nuclease, where a similar truncation was tested (15). For crRNA version –15, which contains the hairpin forming part of the scaffold but cannot form the pseudo-knot structure, we detect efficient target DNA cleavage but the binding of this version to the effector is decreased compared to the full-sized crRNA.

Our data show that a 20-nt spacer RNA, when present at sufficiently high concentrations, induces target cleavage by AsCas12a. The result implies that the scaffold does not play an essential role during the target recognition/cleavage reactions. By strong binding to the effector, the scaffold moiety may either simply increase the affinity of the spacer part of crRNA to the effector or it may induce a conformational change that stabilises the interaction of the spacer part with the binary complex or of the RNA/DNA heteroduplex in the ternary complex. Strong binding of the scaffold to LbCas12a was previously demonstrated and excess of scaffold RNA inhibited DNA cleavage by LbCas12a effector charged with full-sized crRNA (13). Our data show that crRNA scaffold, when present separately, strongly increases the ability of spacer RNA to induce target cleavage by AsCas12a effector. Since in split crRNA the scaffold is physically separated from the spacer, it follows that the binding of the scaffold can not stimulate target recognition/cleavage by simple increase of the local concentration of the spacer. We, therefore, predict that crystallographic analysis of a Cas12a effector bound with a crRNA scaffold shall reveal conformational changes that affect the site that interacts with the spacer.

Despite substantial differences in their amino acid sequences, the Cas12a orthologs tested here have very similar structures of the crRNA scaffold which differ only in the loop region (nucleotides –8–9, Figure 7A) (25). While FnCas12a and AsCas12a scaffolds differ only by a single nucleotide in the –9 position, the LbCas12a scaffold has a distinct loop sequence UAAGU (UCUU and UGUU in AsCas12a and FnCas12a, correspondingly). This loop may contribute to the less efficient binding of LbCas12a charged with split crRNA in the beacon experiments (Figure 7B).

Our data on the split crRNA/Cas12a system may not just help to understand the molecular mechanism of Cas12a better but can be useful for *in vitro* applications of Cas12a in DETECTR-based diagnostics (16). By combining short, 20-nt RNA mixes containing a single scaffold and multiple spacer parts, multiplex recognition of different targets can be achieved at significant cost saving compared to a situation when the same targets have to be probed with individual 40-nt full-sized crRNAs. Further down the road, split crRNAs may be useful in genome editing, however, the problem of delivery and/or stability of very short RNAs would have to be addressed first. The *in vitro* work presented here shows that both sides of the split site in crRNA can be modified without compromising activity, which may be a promising opening to address the stability issue.

## DATA AVAILABILITY

All data presented in the manuscript is available upon request.

## Supplementary Material

gkab1227_Supplemental_File
