# Supplementary material for: Efficient target cleavage by Type V Cas12a effectors programmed with split CRISPR RNA
Source: Nucleic Acids Res. 2021 Dec 24;50(2):1162–73. doi: 10.1093/nar/gkab1227 (PMC8789034; doi:10.1093/nar/gkab1227)
Supplement: gkab1227_Supplemental_File [file gkab1227_supplemental_file.pdf]

SUPPLEMENTARY MATERIALS

Chemical synthesis of RNA

The oligoribonucleotides were synthesized using an automatic DNA/RNA synthesizer ASM-2000 (Biosset Ltd.) at 0.5 μmol scale on 1000 Å Universal support CPG (Primetech ALC, 41 μmol/g). Dichloroacetic acid (3% (v/v) in dichloromethane) was used as the detritylation agent. Coupling step was performed with 5-benzylthio-1H-tetrazole (0.3 M in acetonitrile) and standard 2'-O-TBDMS protected phosphoramidites (0.1 M in acetonitrile) (ChemGenes Corp.). Biotin modification was introduced with commercially available phosphoramidite (Primetech ALC). On oxidation step 0.02 M iodine in a mixture of THF, water, and pyridine was used. Capping was done with CAP A (15 % (v/v) acetic anhydride in THF) and CAP B (16 % (v/v) N-methylimidazole in THF). Oligonucleotides were cleaved and deprotected with ammonium hydroxide/40% aqueous methylamine (1:1 v/v) at 65 °C for 1.5 h. The TBDMS groups were cleaved with NEt_3 3HF (55 % (v/v) in DMSO) solution at 65 °C for 2.5 h. Oligonucleotides were purified by preparative ion-exchange HPLC or by PAGE. IE HPLC was done with a linear gradient from 10 to 60% buffer B for 40 min at a flow rate of 1 mL/min (Agilent 1260 infinity). Buffer A was 50 mM Tris–HCl pH 7.6 in sterile water/acetonitrile (9 : 1 v/v), buffer B - 600 mM $NaClO_4$ and 50 mM Tris–HCl pH 7.6 in sterile water/acetonitrile (9 : 1 v/v). The column was Tosoh Bioscience LLC TSKGEL SUPERQ-5PW 7.5X75 (thermostated at 55 °C). Fractions containing pure oligonucleotides were evaporated and desalted using acetone precipitation. Preparative PAGE was done in 17 % denaturing (6,5 M urea) gells (19:1 acrylamide to bisacrylamide ratio). Oligonucleotides were extracted from gel with 0.5 M NH_4OAc, 1 mM EDTA, pH 8.0, and precipitated with EtOH. Purified oligonucleotides were characterized by UV-photometry, analytical HPLC, and PAGE.

Supplementary Table 1. Sequences of oligonucleotides used in the study

name	sequence
**RNA**	
**crRNA**	UAAUUUCUACUCUUGUAGAUCCCCUCUAUUGAUCCCCACC
**crRNA 2**	UAAUUUCUACUCUUGUAGAUGAGAAGUCAUAUAAUAAGGUA
**crRNA competitor / PAM2 crRNA**	UAAUUUCUACUCUUGUAGAUGCUGUUCCCCAACCUUUUCC
**crRNA-scaffold**	UAAUUUCUACUCUUGUAGAU
**crRNA-spacer**	CCCCUCUAUUGAUCCCCACC
**crRNA 2 spacer**	GAGAAGUCAUAUAAUAAGGUA
**crRNA-scaffold-5bio**	biotin-UAAUUUCUACUCUUGUAGAU
**crRNA-scaffold-3bio**	UAAUUUCUACUCUUGUAGAU-biotin
**crRNA-spacer-5bio**	biotin-CCCCUCUAUUGAUCCCCACC
**crRNA-spacer-3bio**	CCCCUCUAUUGAUCCCCACC-biotin
**“-15” crRNA**	UCUACUCUUGUAGAUCCCCUCUAUUGAUCCCCACC
**“-17” crRNA**	UUUCUACUCUUGUAGAUCCCCUCUAUUGAUCCCCACC

**“-7” crRNA**	UGUAGAUCCCCUCUAUUGAUCCCCACC
**“-2” crRNA / “-2-Spacer”**	AUCCCCUCUAUUGAUCCCCACC
**“+2” crRNA**	UAAUUUCUACUCUUGUAGAUCC
**PAM2 spacer**	GCUGUUCCCCAACCUUUUCC
**Lb crRNA**	UAAUUUCUACUAAGUGUAGAUGAGAAGUCAUAUAAUAAGGUA
**Lb crRNA scaffold**	UAAUUUCUACUAAGUGUAGAU
**Fn crRNA**	UAAUUUCUACUGUUGUAGAUGAGAAGUCAUAUAAUAAGGUA
**Fn crRNA scaffold**	UAAUUUCUACUGUUGUAGAU
**Fn crRNA spacer**	GAGAAGUCAUAUAAUAAGGUA
**sgRNA**	GAGUUUUAUGGCGUCAGCGAGUUUUAGAGCUAGAAAUAGCAAG UUAAAAUAAGGCUAGUCCGUUAUCAACUUGAAAAAGUGGCACC GAGUCGGUGCU
**sgRNA-spacer**	GAGUUUUAUGGCGUCAGCGA
**sgRNA-scaffold**	GUUUUAGAGCUAGAAAUAGCAAGUUAAAAUAAGGCUAGUCCGU UAUCAACUUGAAAAAGUGGCACCGAGUCGGUGCU
**DNMT1 crRNA**	UAAUUUCUACUCUUGUAGAUCUGAUGGUCCAUGUCUGUUA

**DNMT1 crRNA-spacer**	CUGAUGGUCCAUGUCUGUUA
**DNA primers**	
**L7336**	GATTTGAGAAGCCTTCGCTTC
**H11381**	AAGTGGAGTCCGTAAAGAGG
**L2797**	GTCCTAAACTACCAAACCTGC
**H4501**	TGTGCCTGCAAAGATGGTAG
**7831F**	CATCCTTTACATAACAGACG
**13555R**	AGGCGTTTGTGTATGATATGTTTGC
**F DNMT1**	CCACACATGTGAACGGACAGATTGAC
**R DNMT1**	AGCCCGAGAGAGTGCCTCAGGT
**DNA**	
**Target DNA**	5'TACCCGGAGATCGTTTAGAGAAGTCATATAATAAGGTAACTGTTAGAAGCTTGG3' NTS<br>3'ATGGGCCTCTAGCAAATCTCTTCAGTATATTATTCCATTGACAATCTTCGAACC5' TS
**Non-target DNA**	5'TACCCGGAGATCGTTTACTCTTCAGTATATTATTCCATACTGTTAGAAGCTTGG3' NTS<br>3'ATGGGCCTCTAGCAAATGAGAAGTCATATAATAAGGTATGACAATCTTCGAACC5' TS

**Target DNA labeled**	5'TACCCGGAGATCGTTTAGAGAAGTCATATAATAAGGTAACTGTT AGAAGCTTGG3' NTS<br>3'/36FAM/ATGGGCCTCTAGCAAATCTCTTCAGTATATTATTCCATT GACAATCTTCGAACC-5' TS<br>5'/36FAM/TACCCGGAGATCGTTTAGAGAAGTCATATAATAAGGTA ACTGTTAGAAGCTTGG3' NTS<br><br>3'ATGGGCCTCTAGCAAATCTCTTCAGTATATTATTCCATTGACAAT CTTCGAACC5' TS
**Non-target DNA labeled**	5'AGCTTGTCTGCCATGGACATGCAGACTATACTGTTATTGTTGTAC AGACCGAATTCCC/36FAM/3'<br>3'TCGAACAGACGGTACCTGTACGTCTGATATGACAATAACAACAT GTCTGGCTTAAGGG5'<br>5'/56FAM/AGCTTGTCTGCCATGGACATGCAGACTATACTGTTATTG TTGTACAGACCGAATTCCC-'<br>3'TCGAACAGACGGTACCTGTACGTCTGATATGACAATAACAACAT GTCTGGCTTAAGGG5'

The PAM and protospacer/target sequences in DNA oligos are colored in red and blue, respectively. The spacer moieties of RNA oligos are colored in.

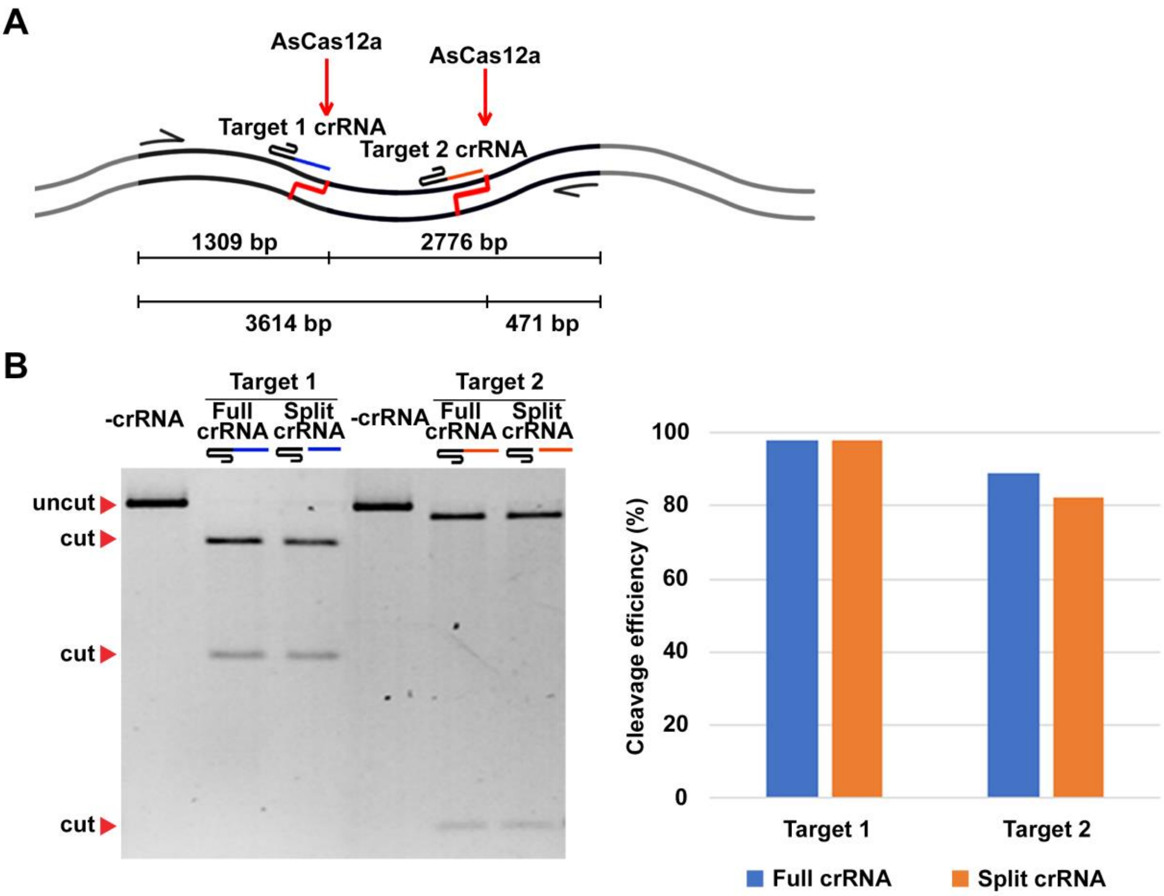

Figure S1. Split crRNA activity is not dependent on the sequence of the crRNA spacer moiety.

(A) Schematic representation of target DNA containing two AsCas12a PAM sequences. Spacer moieties of target crRNAs are shown in blue and orange, the cleavage sites are marked with red arrows. The sizes of the fragments corresponding to cleaved DNA template are given below the scheme for each cleavage site.

(B) *In vitro* cleavage of target DNA by AsCas12a loaded with 500 nM of full-sized or split crRNAs targeting different sites ("Target 1" or "Target 2"", as indicated above the panel). Cleavage efficiencies calculated from the gel presented are shown.

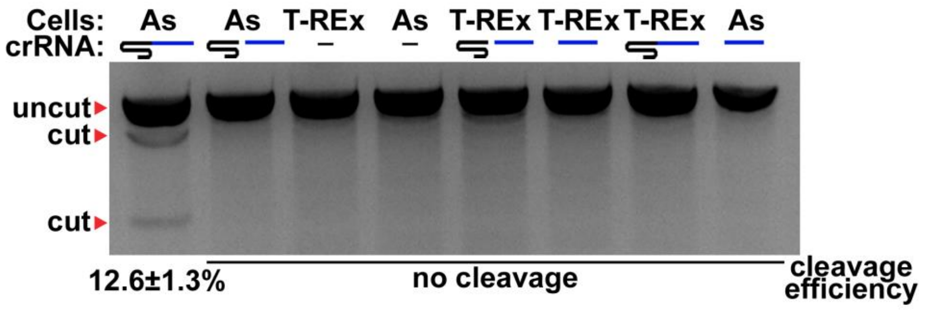

Figure S2. CRISPR/AsCas12a genome editing in HEK293 cells encoding hAsCas12a.

A mismatch-specific T7E1 endonuclease assay was used to analyze DNA flanking AsCas12a target site - DNMT1. HEK293 T-REx hAsCas12a ("As") was transfected with either full-sized or split crRNA (as indicated above the panel) followed by activation of nuclease gene expression. As a control, we used HEK293 T-REx cell line ("T-REx"). Mock transfection was performed to evaluate the effect of the transfection reagent on cells.

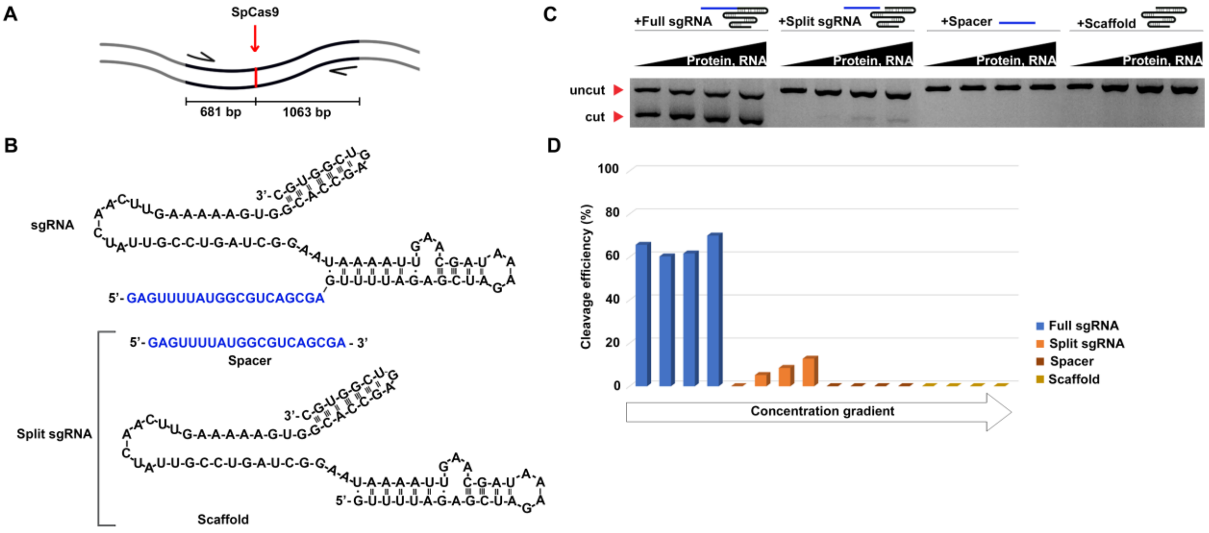

Figure S3. Effect of full-length and split sgRNA versions on SpCas9 cleavage activity.

(A) Schematic representation of the target DNA construct used as a template in the *in vitro* cleavage assay. The black arrows correspond to DNA oligonucleotides used for PCR

amplification of cleavage substrate. The SpCas9 cleavage site is represented with the red arrow. The expected sizes of cleavage products are indicated below the scheme.

(B) Sequence of sgRNAs used. The spacer moiety of the SpCas9 sgRNA is highlighted in blue font and the scaffold moiety is highlighted in black font.

(C) The products of *in vitro* cleavage of the DNA template by purified SpCas9 in the presence of increasing amounts (1, 3, 6, and 8 μM) of indicated sgRNA forms.

(D) Quantification of target DNA cleavage efficiency.
